# Colloidal Cu-Zn-Sn-Te Nanocrystals: Aqueous Synthesis and Raman Spectroscopy Study

**DOI:** 10.3390/nano11112923

**Published:** 2021-10-31

**Authors:** Volodymyr Dzhagan, Olga Kapush, Nazar Mazur, Yevhenii Havryliuk, Mykola I. Danylenko, Serhiy Budzulyak, Volodymyr Yukhymchuk, Mykhailo Valakh, Alexander P. Litvinchuk, Dietrich R. T. Zahn

**Affiliations:** 1 V. Lashkaryov Institute of Semiconductors Physics, National Academy of Sciences of Ukraine, 03028 Kyiv, Ukraine; dzhagan@isp.kiev.ua (V.D.); savchuk-olja@ukr.net (O.K.); nazarmazur@isp.kiev.ua (N.M.); yevhenii.havryliuk@physik.tu-chemnitz.de (Y.H.); buser@ukr.net (S.B.); yukhym@isp.kiev.ua (V.Y.); valakh@isp.kiev.ua (M.V.); 2 Physics Department, Taras Shevchenko National University of Kiev, 01601 Kyiv, Ukraine; 3 Semiconductor Physics, Institute of Physics, Chemnitz University of Technology, 09107 Chemnitz, Germany; 4 Center for Materials, Architectures and Integration of Nanomembranes (MAIN), Chemnitz University of Technology, 09107 Chemnitz, Germany; 5 Frantsevich Institute for Problems of Materials Science, National Academy of Sciences of Ukraine, 03142 Kyiv, Ukraine; myd3@ukr.net; 6 Texas Center for Superconductivity and Department of Physics, University of Houston, Houston, TX 77204-5002, USA; litvin@Central.UH.EDU

**Keywords:** colloidal nanocrystals, Raman spectroscopy, phonons, Cu_2_ZnSnTe_4_, infrared absorber

## Abstract

Cu-Zn-Sn-Te (CZTTe) is an inexpensive quaternary semiconductor that has not been investigated so far, unlike its intensively studied CZTS and CZTSe counterparts, although it may potentially have desirable properties for solar energy conversion, thermoelectric, and other applications. Here, we report on the synthesis of CZTTe nanocrystals (NCs) via an original low-cost, low-temperature colloidal synthesis in water, using a small-molecule stabilizer, thioglycolic acid. The absorption edge at about 0.8–0.9 eV agrees well with the value expected for Cu_2_ZnSnTe_4_, thus suggesting CZTTe to be an affordable alternative for IR photodetectors and solar cells. As the main method of structural characterization multi-wavelength resonant Raman spectroscopy was used complemented by TEM, XRD, XPS as well as UV-vis and IR absorption spectroscopy. The experimental study is supported by first principles density functional calculations of the electronic structure and phonon spectra. Even though the composition of NCs exhibits a noticeable deviation from the Cu_2_ZnSnTe_4_ stoichiometry, a common feature of multinary NCs synthesized in water, the Raman spectra reveal very small widths of the main phonon peak and also multi-phonon scattering processes up to the fourth order. These factors imply a very good crystallinity of the NCs, which is further confirmed by high-resolution TEM.

## 1. Introduction

The growing demand for clean, renewable, and portable energy sources inspires the search for materials with suitable structural characteristics and appropriate parameters of charge generation and transport, thermal transport, and optical properties. A vast playground for tuning the properties for various applications is the family of quaternary chalcogenides I_2_-III-IV-VI_4_ (I = Cu or Ag, II = Zn, Ba, or Cd, IV = Si, Ge, or Sn, and VI = S, Se, or Te) [1,2,3,4,5,6,7,8,9]. It is well known that among binary and ternary chalcogenides the tellurides are generally more promising for thermoelectric applications [10], while the red-shifted bandgap of quaternary telluride compared to sulphides and selenides can be advantageous for photovoltaics [11,12]. However, contrary to intensively investigated sulphides and selenides, as indicated by thousands of publications so far, including numerous reviews [1,2,3,4,5,6,8,9,13,14,15], studies regarding the telluride counterpart were reported only in several works so far [16,17,18,19,20]. In particular, the band structure calculation on a hypothetical compound Cu_4_ZnSn_2_Te_7_ revealed that semiconducting material with a definite bandgap ~0.5 eV could be realized [16]. In Ref. [18], this compound was synthesized by a traditional solid-state tube method and ball-milling, and its thermoelectric (TE) properties were investigated. The thermoelectric properties of bulk Cu_2_ZnSnTe_4_ were investigated in Ref. [19]. The temperature dependence of the electrical properties in In/Cu_2_ZnSnTe_4_/Si/Ag diodes was studied in Ref. [21]. A wide range of Cu_2_-II-IV-Te_4_ compounds (II: Zn, Cd, Hg, and IV: Si, Ge, Sn) was studied with respect to the electronic structure and TE properties in Ref. [22]. There was only one publication, to the best of our knowledge, where colloidal Cu_2_ZnSnTe_4_ NCs were reported (synthesized in hot-boiling solvents) [17]. The scarcity of studies on multinary tellurides is likely to be caused by two critical issues that meet in these compounds. First, there is an extremely small region of stability of quaternary chalcogenides (compared to binaries and ternaries) in the phase diagram [1,2,3,4,5]. The second issue is the precursor of tellurium, which is not available in such convenient and safe form as for sulphur and selenium [2,8,9,14].

Raman spectroscopy has proven to be an indispensable characterization tool in the case of CZTS and CZTSe [23,24,25,26]. While XRD suffers from overlap of the CZTS(e) pattern with those of potential secondary phases, the latter can be reliably distinguished from each other and from the main (quaternary) compound based on the spectrally distinct vibrational patterns in the Raman spectra [24,27,28]. For I-II-IV-VI tellurides, Raman spectra were reported only for bulk stannite Cu_2_CdSnTe_4_ [29], microcrystalline (bulk-like) kesterite Cu_2_ZnSnTe_4_ [30], bulk Cu_2_ZnSiTe_4_ [31,32,33], and Cu_2_ZnGeTe_4_ [34]. Obtaining a Raman spectrum of a new compound in the NC form contributes to fundamental knowledge about phonons in complex compounds and peculiarities of the lattice dynamics under strong spatial confinement in NCs.

Here, we report on an affordable and scalable water-based approach to the synthesis of colloidal CZTTe NCs stabilized by a small-molecule ligand, thioglycolic acid, and perform a detailed structural study of these NCs by means of multi-wavelength resonant Raman spectroscopy, supported by XPS, TEM, XRD, UV-vis and IR spectroscopy, as well as by first principles density functional theory (DFT) calculations.

## 2. Materials and Methods

### 2.1. Materials

Thioglycolic acid (TGA), CuCl_2_ × 2H_2_O, SnCl_2_ × 2H_2_O, Zn(CH_3_COO)_2_ × 2H_2_O, NaOH, NaBH_4_, Te (powder, 99.997% trace metals basis), 2-propanol were supplied by Sigma Aldrich (Burlington, MA, USA). All chemicals were used as received. All glassware was cleaned with aqua regia (3:1 *v*/*v* HCl (37%):HNO_3_ (65%) solutions) and then rinsed thoroughly with deionized water (DI water) before use. Caution: aqua regia solutions are dangerous and should be used with extreme care; never store these solutions in closed containers. Deionized water (18 MΩ cm, Merck Millipore Burlington, MA, USA) was used to prepare all solutions in the experiments.

### 2.2. Synthesis of Cu-Zn-Sn-Te NCs

Colloidal Cu-Zn-Sn-Te nanocrystals (NCs) were obtained by an original low-temperature colloidal synthesis in the presence of TGA as a stabilizer. The synthesis was performed in an Erlenmeyer flask, with continuous stirring, using deionized water as the dispersion medium, and can be divided into three steps: (I) preparation of stock tin precursor solution; (II) preparation of a tellurium precursor solution (NaHTe); (III) injection of NaHTe solution into the cation-TGA solution. The amount of the solvent can be adjusted, e.g., to obtain a certain viscosity needed for film deposition or other applications.

(I) Preparation of the tin precursor stock solution. The tin precursor stock solution was prepared similarly to the procedure described previously in Ref. [35]. A stock aqueous 0.5 M solution of SnCl_2_ in 4.0 M NaOH was prepared by slowly pouring an aqueous 1.0 M suspension of SnCl_2_ × 2H_2_O into an aqueous 8.0 M solution of NaOH (volumic ratio of the suspension and NaOH solutions were 1:1) and was left for 24 h at room temperature.
(II) Preparation of a tellurium precursor solution (NaHTe). It starts with NaBH_4_ reduction of Te to sodium telluride (NaHTe) liberating H_2_ and NaHTe in an aqueous medium, with subsequent dissociation of NaHTe, which releases Te anions (Te^2-^). The NaHTe solution is usually prepared using tellurium (Te) powder and sodium borohydride (NaBH4), which are dissolved in water or another solvent in an inert atmosphere. The reaction of the tellurium precursor can be written according to the equation.

4NaBH_4_(s) + 2Te(s) + 7H_2_O(l) → 2NaHTe(aq) + Na_2_B_4_O_7_(aq) + 14H_2_(g)

The oxidation reaction is rapid at room temperature and the clear mixture immediately changes to black within 1 min.

2NaHTe + O_2_ = 2NaOH + 2Te

In this work, it was possible to avoid the oxidation of NaHTe by oxygen contained in air, because the synthesis is carried out using a pre-cooled aqueous solution in a syringe. This is a very important step that prevents the use of an inert atmosphere or a vacuum reaction. The optimized NaBH_4_ to Te ratio is 5:1. Excess of NaBH_4_ increases the rate of reaction because Te is reduced faster, while equal or lower amounts of NaBH_4_ slow the reaction down. The reduction process begins with a change in color from clear to purple revealing the formation of NaHTe. The synthesis is completed when the color of the solution is no longer changing. Typically, the reaction lasted 1–2 days depending on the volume of the solution, the amount of NaBH_4_, and the ambient temperature. The Te precursor cannot be stored in ambient air as it gets oxidized forming a black precipitate but could be stored in an inert atmosphere.

Colloidal NCs were synthesized in a reaction between the mixture of TGA complexes of Cu^2+^, Zn^2+^, and Sn^2+^, and NaHTe in deionized water. In a typical synthesis, 0.6 mL aqueous 1.0 M CuCl_2_ solution, 0.6 mL aqueous 0.5 M SnCl_2_ with 4.0 M NaOH solution, and 0.3 mL aqueous 1.0 M Zn(CH_3_COO)_2_ solution were consecutively added to 16 mL deionized water under stirring followed by the addition of 0.44 mL TGA and 0.05–0.7 mL aqueous 8.0 M NaOH solution. Finally, 0.6 mL aqueous 1.0 M NaHTe solution was added. The synthesis was complete when the color of the suspension no longer changed. Typically, the reaction took 30–60 s.

Our study showed that the solutions obtained along with CZTTe NCs may contain a stabilizer excess, Cl^-^, CH3COO^2-^ ions, and (depending on the ratio of initial compounds) Na^+^, Cu^2+^, Zn^2+^, and Sn^2+^ ions as well as highly dispersed crystalline tellurium. Taking into account that CZTTe NCs obtained by this procedure have a charge on the surface, the ions present in the system can interact with the NC surfaces. To decrease the effect of the nature and amount of precursors on the stability and the optical properties of the colloidal solutions obtained, we developed a procedure for the recovery of CZTTe NCs from the parental solutions by a selective precipitation method similar to the one in our previous report on CdTe NCs [36]. For this purpose, equimolar volumes of the initial solution and 2-propanol were mixed in a glass cylinder and kept for 1 min at room temperature under constant stirring. Flocculation of particles occurs because TGA-stabilized NCs are insoluble in 2-propanol. When a lower amount of 2-propanol is added, no flocculation is observed, while an excess of 2-propanol degrades the thioglycolate shell of the NCs and therefore the solution loses its sedimentation stability. The obtained mixture was centrifuged for 1–5 min at a rate of 2000–10,000 rpm. The obtained floccula were separated from the mother liquor by decantation, washed many times with 2-propanol, and peptized in deionized water.

For the spectroscopic studies, the freshly synthesized and purified samples were drop-casted on cleaned substrates, bare Si(100) for Raman and XRD, Au/Si for XPS, and placed for drying in a desiccator under dynamic vacuum. Dry samples showed better chemical stability and reproducible Raman spectra were obtained after (at least) one month of sample storage.

### 2.3. Characterization

XRD patterns were taken with a Rigaku SmartLab X-ray diffractometer (Rigaku Europe SE, Neu-Isenburg, Germany) equipped with a Ni filtered Cu Kα X-ray source. The measurements were performed in θ–2θ geometry with a step of 0.05° 2θ and acquisition speed of 5°/min. The sharp lines from the Si(100) substrate were identified and subtracted.

Raman spectra were excited using 514.7, 532, 638, or 785 nm solid state lasers, 633 nm He-Ne laser, or 325 nm He-Cd laser and registered with a spectral resolution of about 2 cm^−1^ for visible and 5 cm^−1^ for UV excitation using a LabRam HR800 or Xplora (HORIBA Jobin Yvon GmbH, Bensheim, Germany) micro-Raman systems equipped with cooled CCD detectors. The incident laser power under the microscope objective (50×) was 0.1–0.01 mW.

XPS measurements were performed with an ESCALAB 250Xi X-ray Photoelectron Spectrometer Microprobe (Thermo Scientific, Waltham, MA, USA) equipped with a monochromatic Al Kα (hν = 1486.68 eV) X-ray source. A pass energy of 200 eV was used for survey spectra, 40 eV for Auger spectra, and 20 eV for high-resolution core-level spectra (providing a spectral resolution of 0.6 eV). Spectra deconvolution and quantification were performed using the Avantage Data System (Thermo Scientific, Waltham, MA, USA). The linearity of the energy scale was calibrated by the positions of the Fermi edge at 0.00 ± 0.05 eV, Au4f_7/2_ at 83.95 eV, Ag3d_5/2_ at 368.20 eV, and Cu2p_3/2_ at 932.60 eV measured on in situ cleaned metal surfaces. To prevent charging, the NCs samples were measured using a built-in charge compensation system. Finally, the spectra were corrected to the C1s sp^3^ peak at 284.8 eV as a common internal standard for binding energy (BE) calibration [37].

Density functional calculations of the electronic ground state of kesterite CZTTe were performed within the generalized gradient approximation using the Perdew–Burke–Ernzerhof local functional [38] as implemented in the CASTEP software package [39]. Norm-conserving pseudopotentials were used. The plane wave basis set cut-off was set to 800 eV. Before performing calculations, the structure was relaxed and forces on atoms in the equilibrium positions did not exceed 2 meV/Å and the residual stress was below 0.01 GPa. Sampling over the Brillouin zone was performed over a 3 × 3 × 4 Monkhorst-Pack grid in reciprocal space. CZTTe is clearly identified as a direct bandgap semiconductor with the minimum of the conduction band and the maximum of the valence band situated at the Brillouin zone center.

As the next step, the vibrational properties of CZTTe were accessed via density functional perturbation theory, which takes into account small atomic displacements as perturbations. Furthermore, considering the effect of an electric field as perturbation, it also allows the effect of long range electrostatic interactions to be taken into account, which provides the transverse-longitudinal optical (TO-LO) splitting of polar modes in the crystal. It was demonstrated earlier that this computational approach produces very reliable results in describing the vibrational spectra of quaternary semiconductors of various crystallographic structures [6,13,40].

## 3. Results and Discussion

In the general case of the synthesis of Cu-Sn-Zn-Te NCs in solution, each constituent sort of atom is introduced into the reacting vessel in the form of a precursor. The latter is a molecule or a complex, which contains one or more sorts of atoms needed to build the NC. In our case, the source of Cu^2+^ ions was a solution of Cu(NO_3_)_2_, of Sn^2+^ a solution of SnCl_2_ with 4.0 M NaOH, the source of Zn^2+^ ions was Zn(CH_3_COO)_2_, and Te_2_ ions were provided by NaHTe. NCs were stabilized in the aqueous colloid by using thioglycolic acid (TGA). In the course of the reaction that formed Cu-Sn-Zn-Te NCs stabilized with TGA, the color of the solution changes from grey to black.

The synthesis of binary and ternary tellurides reported so far mostly used H_2_Te gas as tellurium precursor [12,36]. However, this gas is extremely toxic. Therefore, using this precursor requires not only a complex and expensive electrochemical cell, in which the gas is produced, but also an additional thermostat and inert gas cylinders to prevent the release of the toxic precursor into the environment. Therefore, a trend can be observed in the field of synthesis to use so-called one pot synthesis [8,9,12,14,26]. However, all of them allow tellurium precursors to be obtained only in an argon atmosphere. This paper shows for the first time the possibility of obtaining NaHTe without the use of inert gas. This markedly simplifies the synthesis and reduces its cost. Since the process of obtaining the Te precursor takes place in a syringe, it is not necessary to use any additional equipment and there is no spontaneous release of the precursor into the environment. The amount of precursor required for one synthesis is synthesized at one time, so there is no excess or waste material. Therefore, the proposed method of synthesis is simpler, cheaper, and more environmentally friendly compared to other methods of obtaining tellurium precursors. Moreover, it allowed Cu-Sn-Zn-Te NCs to be obtained for the first time in a very facile way.

**Figure 1:**
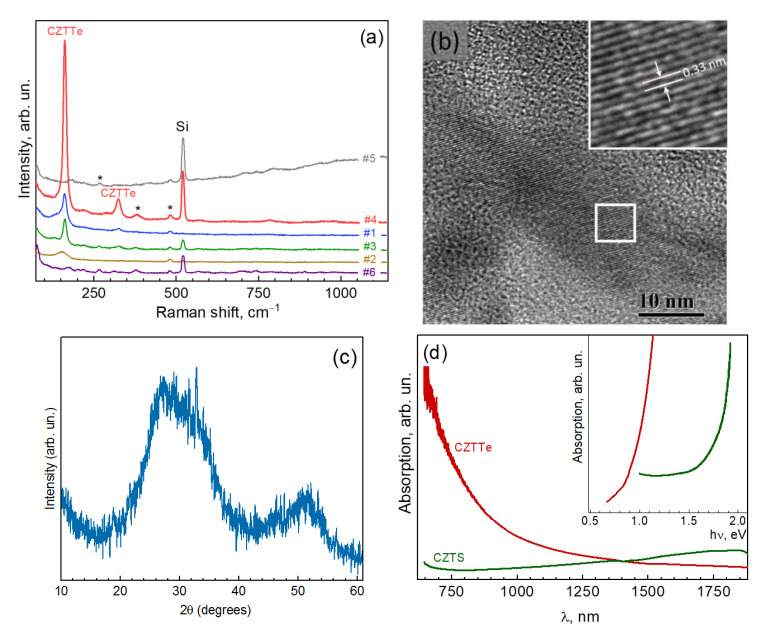
(**a**) Raman spectra (λ_exc_ = 514.7 nm) of a series of NC samples synthesized at varied synthesis conditions, as summarized in Table 1. The peaks denoted with asterisks are not related to the NCs but to organic residuals of synthesis. Representative TEM images (**b**), XRD pattern (**c**), and IR absorption spectrum (**d**) of the CZTTe NCs obtained in this work.

Figure 1a shows Raman spectra of a series of NC samples synthesized at varied synthesis conditions, as summarized in Table 1.

As can be seen from Figure 1a three of the samples exhibit sharp peaks at about 160 and 320 cm^−1^ that differ from the peak of Si substrate and from those of the by-products of the synthesis (marked by asterisks), and can thus be preliminarily assigned to the targeted CZTTe phase. The presence of highly crystalline nanocrystals in the size range of tens of nm in the samples was proved by TEM (Figure 1b). Surprisingly, the XRD patterns of the NC samples are very broad (Figure 1c). This can be attributed to the non-stoichiometry of the NCs (revealed in the XPS spectra analyzed below), as well as by a relatively large fraction of ultrasmall NCs found in most of the samples (Figure S1, Supplementary Materials). The absorption edge at about 0.8–0.9 eV (Figure 1d) agrees well with the value expected for Cu_2_ZnSnTe_4_ [17,30]. Furthermore, in view of a high probability of the formation of secondary phases established earlier for CZTS and CZTSe [27,28,41,42], a possible origin of the peaks at 160 and 320 cm^−1^ stemming from corresponding secondary phases of CZTTe should be examined. Indeed, no peaks corresponding to ZnTe (205 cm^−1^) [43,44,45], Cu_2−x_Te (120–130 cm^−1^) [46], Cu_2_SnTe_3_ (76, 115, 123, 142, and 190 cm^−1^) [47], CuO (290, 345, and 630 cm^−1^) [48,49], or Cu_2_O (218 cm^−1^) [50] are observed in the Raman spectra of the CZTTe NCs in Figure 1a.

Due to the presence of a sulphur-containing stabilizer (thioglycolic acid) in the synthesis medium, the formation of sulphides should not be excluded. For instance, TGA forms a thin CdS shell on CdSe NCs of a small size [51]. However, none of the potential sulphides has its (main or single) Raman peak at about 160 cm^−1^ (see Ref. [28] and refs therein). No ZnS or ZnO was detected by applying resonant UV excitation with λ_exc_ = 325 nm (Figure S2), which is known to be efficient in detecting minor contents of ZnS or ZnO secondary phases in CZTS and other sulphides [24,52].

**Figure 2:**
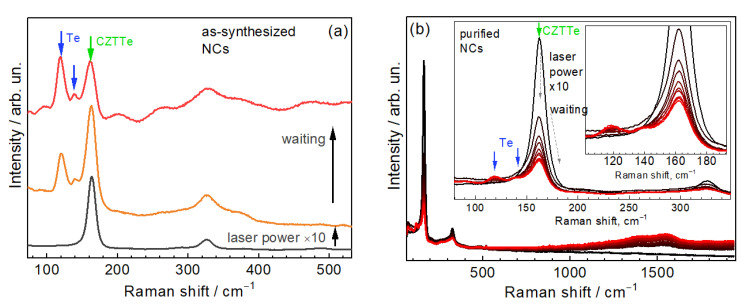
(**a**) Raman spectra (λ_exc_ = 532 nm) of as-synthesized CZTTe NCs (sample #1) at a moderate laser power (bottom curve), at elevated laser power (middle curve), and after illumination for a few minutes at elevated power (upper curve). The appearance of peaks due to photo-induced Te is marked by blue arrows. (**b**) Evolution of the Raman spectra of the same NCs after a purification procedure (see text for detail).

Importantly, elemental tellurium must always be carefully checked as a possible origin of Raman peaks in the spectra of tellurides, as it may occur in the form of nanocrystalline inclusions, few-atom clusters, or amorphous phase [53,54,55,56,57,58]. Due to the huge Raman cross-section of elemental Te, even tiny inclusions or monolayers on the surface of bulk telluride can result in a Raman peak intensity comparable to that of the main (telluride) phase [59]. However, the characteristic Raman peaks of elemental Te of different forms and modifications are well known (120 and 140 cm^−1^) [53,54,55,56,57,58,59,60] and these are not observed in our spectrum. Only after intense laser irradiation of the sample, which leads to the decomposition of the NCs (Figure 2a), the spectral features of elemental Te become detectable in the Raman spectra. The peak position of amorphous Te (a-Te), ~160 cm^−1^ [60,61,62], matches the position of the main Raman peak of CZTTe NCs in the present study. However, the feature of a-Te is considerably broader and is not expected to produce multiple overtones that are characteristic of a crystalline semiconductor lattice [61]. The attribution of the 160 cm^−1^ mode to CZTTe is furthermore corroborated by this peak being preserved in the spectrum (Figure 2b) of the NC sample after removal of excess (free) ligands and residuals of the synthesis such as salts, hydroxides, etc. (see Experimental). Noteworthy is that the photoinduced formation of elemental Te is strongly suppressed for purified samples (compare Figure 2a,b). This may be due to the removal of the source of Te itself or the removal of a certain compound that catalyzes the process of its photoinduced crystallization or reduction. Establishing the exact reason may need an extensive additional study.

**Figure 3:**
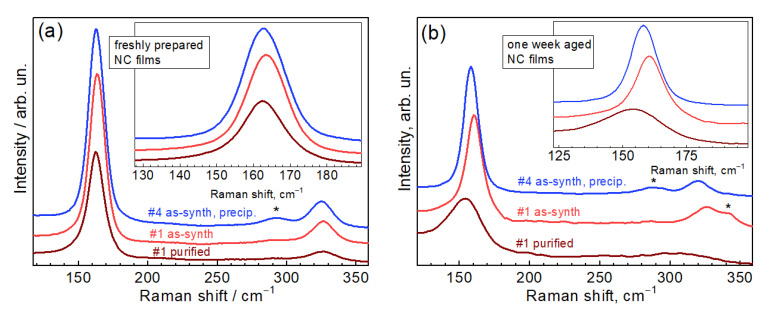
(**a**) Raman spectra (λ_exc_ = 514.7 nm) of freshly prepared films of as-synthesized CZTTe NCs (sample #1), same NCs after purification, and of the precipitate of another as-synthesized NC colloid (#4). (**b**) Spectra of the same film samples after one week of storage in air. The asterisks indicate the peaks originating from residuals of the synthesis.

An additional argument for the assignment of the observed Raman modes to lattice phonons of CZTTe NCs is provided by a comparative analysis of the spectra of as-synthesized and purified NCs, as well as from observing the long-term stability of these two samples. The spectra of the NCs from the as-synthesized and purified sample (#1) reveal identical spectra (Figure 3a), which also coincide with the spectrum of a precipitate of another NC sample (#4) that lost its colloidal stability by itself. When measuring the same three NC films after one week of storage in air, no significant changes occurred compared to the as-synthesized #1 and precipitated #4, while for the purified sample #1 the intensity dropped and the peak got broader and shifted downwards (Figure 3b). These observations can only be explained by partial degradation of the purified NCs that were deprived of additional protection against oxidation. Note that the substantial broadening of the Raman peak with time (Figure 3b), the different full width at half maximum (FWHM) in the spectra of different as-synthesized samples (Figure 1a), as well as some low-frequency asymmetry of the peak are indications of phonon confinement [63], thus proving the origin of this peak to be from CZTTe NCs. Finally, the NCs synthesized under different conditions (Table 1) reveal not only different lineshapes of the main Raman peak, which is very reasonable behavior for NCs but not expected for molecular species, but also differences in photoinduced Te formation, in particular at different λ_exc_ (Figure S3).

In analogy to CZTS and CZTSe, the XRD peaks of corresponding ternary and binary secondary phases, such as Cu_2_SnTe_3_ and ZnTe, cannot be distinguished from those of CZTTe and thus the importance of Raman spectroscopy for phase confirmation for CZTTe is as high as for other quaternary chalcogenides. Despite very distinct and sharp XRD patterns of microcrystalline (bulk-like) kesterite Cu_2_ZnSnTe_4_, very broad Raman features at 122 and 137 cm^−1^ were observed in Ref. [30]. The authors claimed that those peaks are due to the fully symmetric A-modes of CZTTe phonons based on an estimation of the Raman phonon frequencies in CZTTe using a tetra-atomic linear chain model [64] and the Raman frequencies of Cu_2_ZnSiTe_4_ reported earlier [31,32]. These two peaks are, as we already mentioned, very close in position to the known characteristic modes of elemental Te at 120 and 140 cm^−1^ and they were observed in situ in our experiment by irradiating CZTTe NCs at elevated laser powers (Figure 2). Therefore, most likely the spectrum reported in Ref. [30] is not characteristic for CZTTe NCs but indicates the presence of elemental Te.

**Figure 4:**
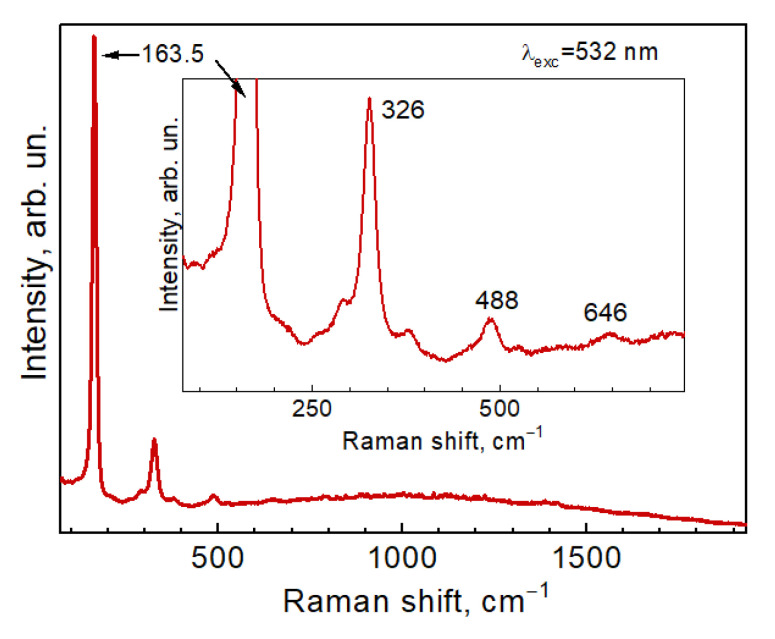
A representative Raman spectrum of CZTTe NCs (sample #1) in a broad spectral range and with zoomed part of the spectrum showing three overtones of the main peak.

The results of our DFT lattice dynamics calculations of CZTTe provide deeper insight into the origin of its “main Raman peak”. As the lattice dynamics properties of quaternary compounds with kesterite structure are well understood by now, including the vibrational patterns of specific optical modes (see, e.g., [40,65,66]), we just mention that the three fully symmetric (A-symmetry) modes of CZTTe are predicted to be at 129, 117, and 113 cm^−1^, much lower in comparison with those of the respective Se and S compounds [17,24,28]. However, similar to sulfides and selenides, several modes with frequencies exceeding those of the A-modes are expected also in CZTTe: B-symmetry transverse optical (TO) vibrations (144, 154, 173 cm^−1^) and E-symmetry in-plane modes (134, 141, 152, and 169 cm^−1^). Importantly, these latter modes are polar, i.e., exhibit transverse-longitudinal (TO-LO) splitting due to long-range electrostatic interactions. Thus, taking into account the occurrence of a remarkable multi-phonon scattering process, as shown in Figure 4, one of the B-symmetry modes (with calculated LO components at 152 and 155 cm^−1^) are good candidates to be the origin of the experimentally observed features.

It was well established in earlier Raman investigations of multinary metal chalcogenides with kesterite structure, that the overtones can be observed not only for the “main” non-polar A-symmetry modes [35] (due to deformation potential interactions) but also for longitudinal optical (LO) modes of polar B-symmetry [67,68], due to Fröhlich-induced interaction. Moreover, a higher order combination of these modes could be observed experimentally [30]. It appears that the fundamental difference of CZTTe in comparison with its Se and S counterparts is due to the much smaller band gap of the former compound and peculiar resonant conditions, which are reflected in a very specific resonant Raman scattering spectrum. Excitation of CZTTe NCs with different λ_exc_, namely 514.7, 633, and 785 nm, did not reveal noticeable changes in the lineshape, although some peak shift and an asymmetry can be seen under red excitation compared to the green one (Figure S6).

**Figure 5:**
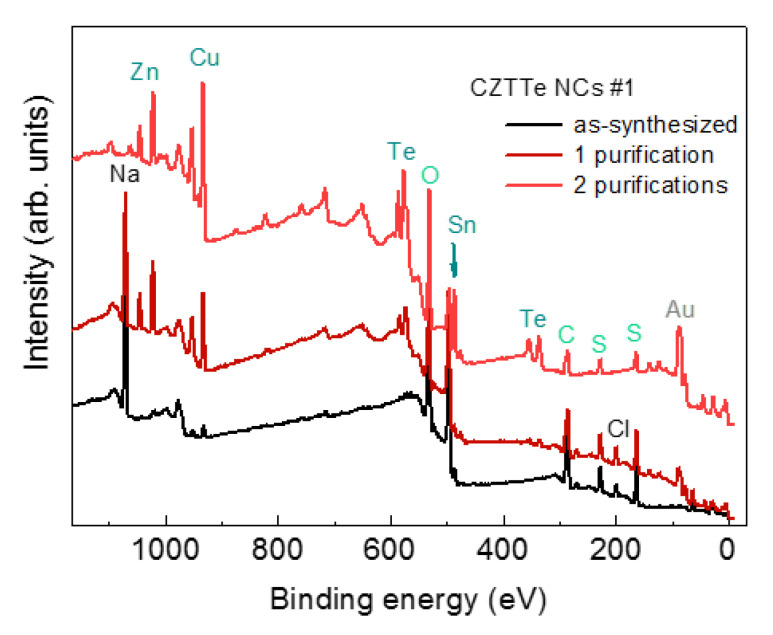
Survey XPS spectra of CZTTe NCs (sample #1) for the as-synthesized sample and samples subjected to one and two purification cycles. The Au signal stems from the substrate used to deposit NCs for XPS measurements.

Elemental analysis of as-synthesized CZTTe NCs by XPS revealed the dominating contribution of C, O, S, and Na, with hardly any traces of the NC elements (Figure 5, bottom curve), which is a common situation for this type of colloidal synthesis that uses significant excess of the stabilizer to ensure the formation of NCs in water. After a standard purification procedure, the peaks stemming from the elements in the NCs gain in intensity (middle curve) and become dominant after repeated purification (upper curve).

**Figure 6:**
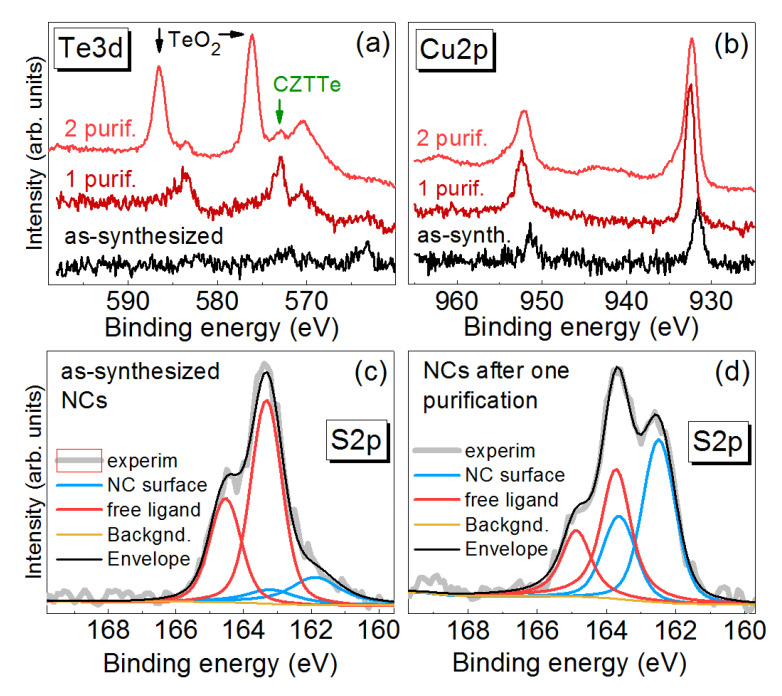
High-resolution Te3d (**a**) and Cu2p (**b**) XPS spectra of CZTTe NCs (sample #1) for as-synthesized NCs and those subjected to one and two purification cycles (upper graphs). The two graphs at the bottom show a comparative fit of the S2p spectra of as-synthesized (**c**) and purified (**d**) NCs, which confirms the removal of free ligands by purification.

In more detail, the effect of purification can be analyzed from the high-resolution XPS spectra of the elements (Figure 6). In particular, no NC-related peak is seen in the Te3d spectrum of the as-synthesized samples. The spectrum after the first purification reveals a distinct doublet stemming from NCs. The second purification step apparently removes too much of the protecting ligand shell from the NCs and thus results in strong oxidation of surface Te to TeO_2_. Note that the NCs synthesized at different conditions show different effects of the purification in this respect, as can be seen from a comparison of the Te3d spectra of NC samples #1, #3, and #4 (Figure S5a). The effect of the purification on the state of NC stabilization by the ligand (thioglycolic acid, TGA) is well seen in the S2p spectra (Figure 6). Thus the ratio of the free TGA molecules to those bound to the NC surface decreases dramatically after the first purification step in agreement with results on other NCs synthesized by a similar route [28]. The second purification step changes this ratio less significantly, but decreases the absolute intensity of both components (seen as a lower signal to noise ratio in the normalized spectra in Figure S5b), indicating the loss of ligands bound to the NC surface. This observation explains the oxidation of surface Te after the second purification (discussed above).

The S2p spectra deliver further very important structural information about the NCs obtained in this work, which can be hardly obtained by other methods. Our recent studies of sulphide NCs stabilized by thiols demonstrated that Sulphur atoms that are a part of the NC lattice (i.e., S^2–^) can be identified by a separate component in the S2p spectra, with a binding energy (BE) for S2p_3/2_ of 160.5–161.5 eV [51,69]. Surprisingly, the presence of a thick sulphide shell was inferred from the XPS S2p and Raman spectra also for selenide NCs synthesized similarly [51,63]. For the present CZTTe NCs, however, no S^2–^ component is detected in the spectra even after the second purification step, indicating that the formation of a sulphide overlayer using thiol Sulphur ions is not likely for the multinary telluride.

**Figure 7:**
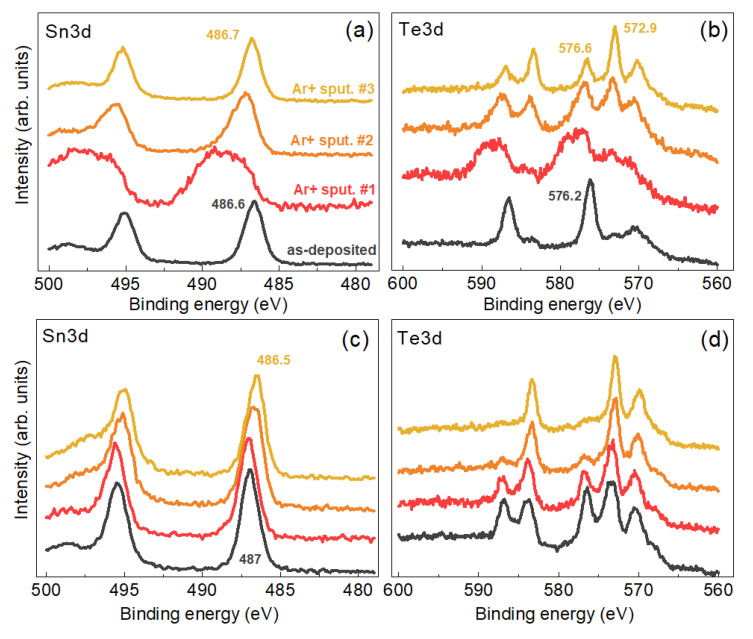
Evolution of the Sn3d (**a**,**c**) and Te3d (**b**,**d**) XPS spectra of CZTTe NCs sample #1 (**a**,**b**) and #4 (**c**,**d**) after subsequent 30 sec sputtering with Ar^+^ ions in three regimes–low flux of 200 eV ions (“Ar+ sput #1”), with medium intensity flux of 500 eV ions (“Ar^+^ sput #2”), and with high-intensity flux of 1000 eV ions (“Ar^+^ sput #3”).

The surface layer of TeO_2_ detected on doubly purified NCs is of sub-nm thickness and can be removed by a soft sputtering in situ in the XPS vacuum chamber. Figure 7 shows the effect of a subsequent 30 sec sputtering with Ar^+^ ions in three regimes—with a low flux of 200 eV ions (#1), with a medium intensity flux of 500 eV ions (#2), and with a high intensity flux of 1000 eV ions (#2). The Te3d BE of 572.9 eV, observed for the sputtered samples, lies between the values reported for elemental Te and Te in a compound with metals [68]. The reduction of Te during sputtering of its oxides with ions is a well-established fact in the literature [70]. Therefore, we can conclude that during the sputtering of the oxidized surface of the CZTTe NCs we partially obtain a Te reduction rather than a complete removal of the oxide layer and exposure of the bare/clean CZTTe NC surface. Note, however, that the effect of non-stoichiometry and the corresponding effect of mixed valency [16] can be responsible for a wider variation of the BE of the elements in the NCs compared to that of the stoichiometric surfaces of bulk samples. In Ref. [16] the 3d_5/2_ BE of 572.8 eV was attributed in Te^2−^ in Cu_5_Sn_2_Te_7_, suggesting that there is no mixed valency of Te^2−^ and Te^1−^. The Sn3d spectra (BE of 3d_5/2_ of 486.5 eV) revealed Sn^4+^ as the main oxidation state of tin in this compound, with only a minor contribution of Sn^2+^ (5%) attributed to SnTe as a secondary phase. For 5 nm CuZn_2_InTe_4_ NC, Te3d_5/2_ was observed at 572.8 eV [71].

In view of the XPS results, the determination of the elemental composition of the present CZTTe NCs is rather challenging, as was already noticed earlier for NCs of other multi-component systems, e.g., CZTS [28]. As XPS is a very surface sensitive technique, XPS spectra stem from the top nanometers of the NC film, with an exponentially decaying contribution from deeper thicknesses. In the case of as-synthesized NCs (without purification), the presence of excess ligands and other residuals of the synthesis precludes observing the NC peaks and thus quantitative analysis (Figure 5, bottom curve). The single purification step leads to strong enough XPS signals of all the elements except that of Tin (Figure 5, middle curve). After the second purification step, also a distinct signal of Tin is observed (Figure 5, upper curve), but the NC composition determined from the XPS can be affected by partial oxidation of the NC surface (Figure 6, upper graphs). Still, we can confidently make the following conclusions on the NC composition derived from the XPS data (in at. %, ±3): Cu:Zn:Sn:Te:S = 35:17:9:9:30. First, the NCs are quite non-stoichiometric, similar to CZTS, CTS, CTSe, and other compounds synthesized by colloidal chemistry in water [28,72]. Nevertheless, the deficit of Sn is less pronounced here than for the CZTS NC counterpart synthesized recently by a similar method [28,35]. Apparently, the growth of CZTTe NCs occurs in chemically different conditions that are more favorable for embedding more Sn into the lattice. Even though, as follows from the XPS data discussed above, Tin is obviously not homogeneously distributed within the NC, presumably leading to a Sn-poor outer part of the NC.

## 4. Conclusions

A route for the synthesis of Cu-Zn-Sn-Te (CZTTe) NCs by an original low-cost method based on a low-temperature colloidal synthesis in water, using a small-molecule stabilizer, thioglycolic acid (TGA), is proposed. Multi-wavelength resonant Raman spectroscopy was employed as the main method of structural characterization, supported by TEM, XRD, XPS, and NIR spectroscopy. The strongest mode in the experimental Raman spectrum at 160 cm^−1^ is shown to be characteristic for CZTTe NCs and assigned to a B-symmetry polar phonon mode. The NC composition strongly deviates from the Cu_2_ZnSnTe_4_ stoichiometry, as determined by XPS. This is common for multinary NCs synthesized in water. Nevertheless, the Raman spectra exhibit a markedly narrow width of the main phonon peak as well as higher order scattering processes up to the fourth order. Both factors are strong indications of good crystal quality as confirmed by high-resolution TEM results.

## Figures and Tables

**Table 1:** Varied synthesis parameters of the CZTTe NCs.

Sample #	(NaOH), mL	Te Precursor	Nominal Amount of Te Precursor
1	0.64	NaHTe	stoichiometric
2	0.1		
3	0.64	NaHTe	in deficit
4	0.1		
5	0.64	Te + NaBH4	in excess
6	0.1		

## Data Availability

The data presented in this study are available on request from the corresponding author.

## Supplementary Materials

The following are available online at https://www.mdpi.com/article/10.3390/nano11112923/s1, Figure S1: TEM images, Figure S2: Raman spectra of CZTTe NCs at λ_exc_ = 325 nm, Figure S3: Raman spectra of CZTTe NCs at different laser powers of λ_exc_ = 785 nm, Figure S4: Raman spectra CZTTe NCs at λ_exc_ = 514.7 nm, Figure S5: XPS spectra of Te 3d spectra of twice purified NC samples #1, #3, and #4, and S2p spectra of NC sample #1 before and after purification steps, Figure S6: A comparative Raman plot for CZTTe NCs (sample #1) at two excitations and different laser powers.
