# Supplementary material for: Colloidal Cu-Zn-Sn-Te Nanocrystals: Aqueous Synthesis and Raman Spectroscopy Study
Source: Nanomaterials (Basel). 2021 Oct 31;11(11):2923. doi: 10.3390/nano11112923 (PMC8624267; doi:10.3390/nano11112923)
Supplement: Supplementary file 1 [file nanomaterials-11-02923-s001.zip › nanomaterials-1407914-supplementary.pdf]

Supplementary Materials

Article

Colloidal Cu-Zn-Sn-Te Nanocrystals: Aqueous Synthesis and Raman Spectroscopy Study

Volodymyr Dzhagan [1,2], Olga Kapush [1], Nazar Mazur [1], Yevhenii Havryliuk [1,3,4], Mykola I. Danylenko [5], Serhiy Budzulyak [1], Volodymyr Yukhymchuk [1], Mykhailo Valakh [1], Alexander P. Litvinchuk [6] and Dietrich R. T. Zahn [3,4,*]

[1] V. Lashkaryov Institute of Semiconductors Physics, National Academy of Sciences of Ukraine, 03028 Kyiv, Ukraine; dzhagan@isp.kiev.ua (V.D.); savchuk-olja@ukr.net (O.K.); nazarmazur@isp.kiev.ua (N.M.); yevhenii.havryliuk@physik.tu-chemnitz.de (Y.H.); buser@ukr.net (S.B.); yukhym@isp.kiev.ua (V.Y.); valakh@isp.kiev.ua (M.V.)

[2] Physics Department, Taras Shevchenko National University of Kiev, 01601 Kyiv, Ukraine

[3] Semiconductor Physics, Institute of Physics, Chemnitz University of Technology, 09107 Chemnitz, Germany

[4] Center for Materials, Architectures and Integration of Nanomembranes (MAIN), Chemnitz University of Technology, 09107 Chemnitz, Germany

[5] Frantsevich Institute for Problems of Materials Science, National Academy of Sciences of Ukraine, Kyiv, Ukraine; myd3@ukr.net

[6] Texas Center for Superconductivity and Department of Physics, University of Houston, Houston, TX 77204-5002, USA; litvin@Central.UH.EDU

* Correspondence: zahn@physik.tu-chemnitz.de

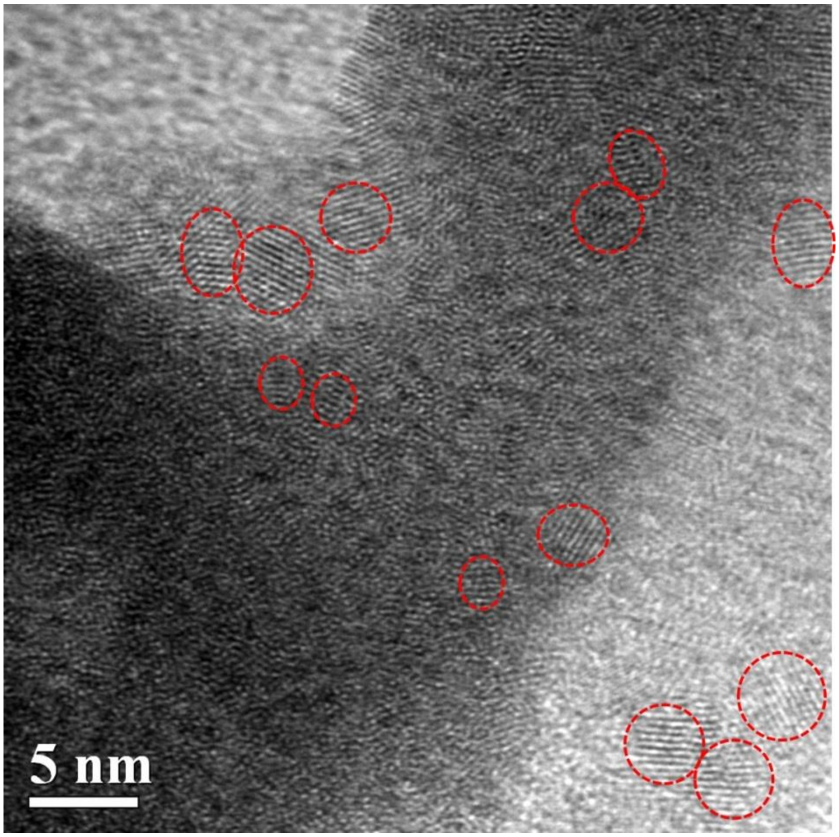

Figure S1. TEM images showing co-existence of regular-size NCs and ultrasmall crystalline clusters.

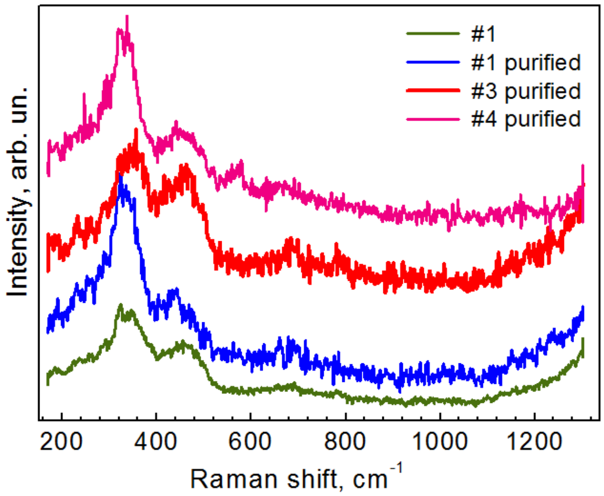

Figure S2. Raman spectra of CZTTe NCs at λ_{exc} = 325nm. No sharp peaks corresponding to pure ZnS (350 cm^{-1}) or ZnO (580 cm^{-1}) are detected. The observed feature at about 330–340 cm^{-1} may be related to Zn-S-like vibrations in the CZTTe NC lattice. The origin of the feature at about 450–470 cm^{-1} needs further investigations.

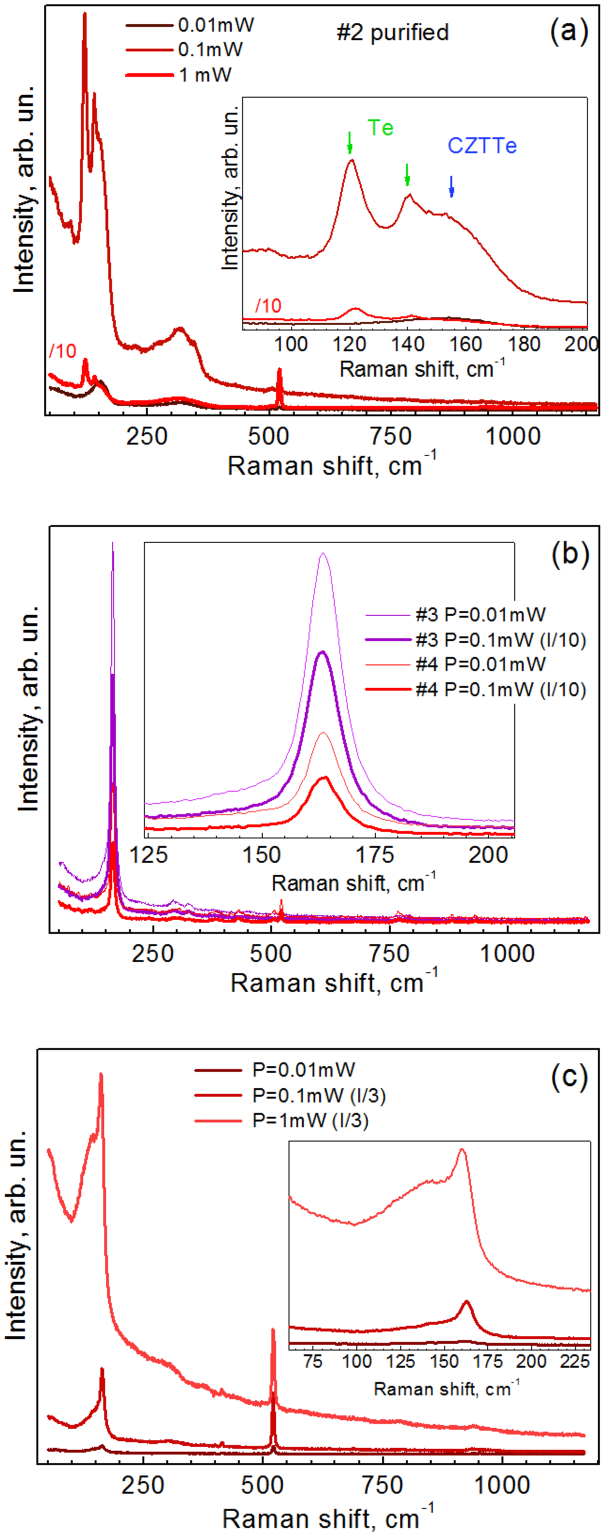

Figure S3. Raman spectra of CZTTe NCs at different laser powers of λ_{exc} = 785 nm: (**a**) sample #2 after single purification step, (**b**) single-purified #3 and #4; (**c**) initial (not purified) #1. The intensity in some spectra is divided by a factor of 3 or 10.

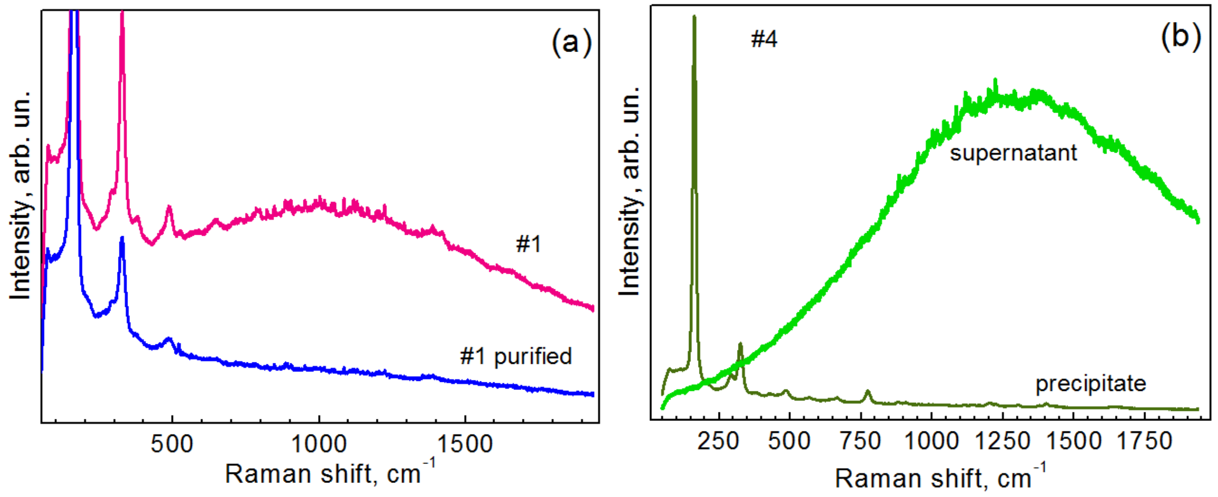

Figure S4. (**a**) Raman spectra CZTTe NCs at λ_{exc} = 514.7 nm :(**a**) sample #1, (**b**) sample #4. The spectra show that the origin of the broad band in the spectra of the initial samples is related with some (minor) byproducts of the synthesis contained in the solution.

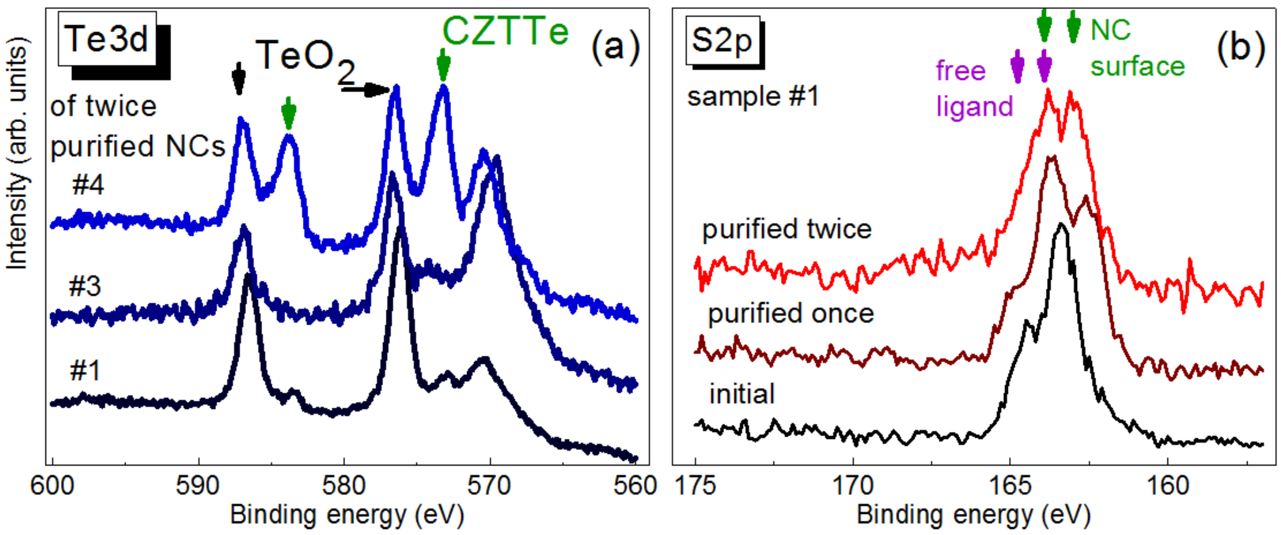

Figure S5. (**a**) XPS spectra of Te 3d spectra of twice purified NC samples #1, #3, and #4, demonstrating better stability of #4 towards oxidation. (**b**) S2p spectra of NC sample #1 as-synthesized (initial), and after one and two purification steps. The spectra demonstrate reduction of the contribution of free ligands and increase of the intensity related with surface bound TGA molecules.

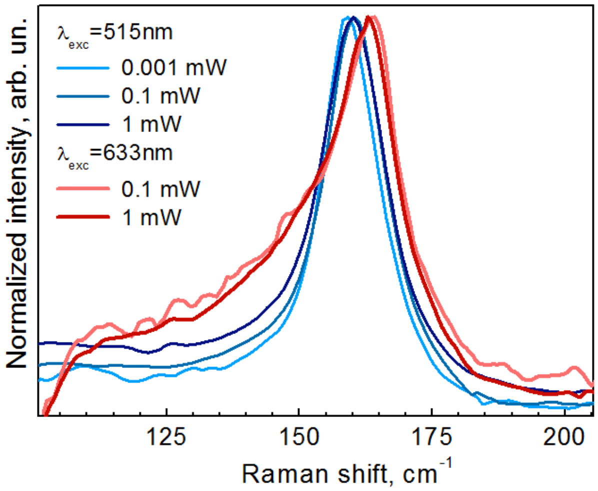

Figure S6. A comparative Raman plot for CZTTe NCs (sample #1) at two excitations and different laser powers.
